# A multidisciplinary approach of workload assessment in real-job situations: investigation in the field of aerospace activities

**DOI:** 10.3389/fpsyg.2014.00964

**Published:** 2014-09-03

**Authors:** Claudine Mélan, Nadine Cascino

**Affiliations:** Laboratory Cognition, Langues, Langage, Ergonomie – Laboratoire Travail et Cognition – UMR 5263, Department of Psychology, University of ToulouseToulouse, France

**Keywords:** workload, job-perception, load factors, alertness, multidisciplinary approach, aerospace activities

## Abstract

The present contribution presents two field studies combining tools and methods from cognitive psychology and from occupational psychology in order to perform a thorough investigation of workload in employees. Cognitive load theory proposes to distinguish different load categories of working memory, in a context of instruction. Intrinsic load is inherent to the task, extraneous load refers to components of a learning environment that may be modified to reduce total load, and germane load enables schemas construction and thus efficient learning. We showed previously that this theoretical framework may be successfully extended to working memory tasks in non-instructional designs. Other theoretical models, issued from the field of occupational psychology, account for an individual’s perception of work demands or requirements in the context of different psychosocial features of the (work) environment. Combining these approaches is difficult as workload assessment by job-perception questionnaires explore an individual’s overall job-perception over a large time-period, whereas cognitive load investigations in working memory tasks are typically performed within short time-periods. We proposed an original methodology enabling investigation of workload and load factors in a comparable time-frame. We report two field studies investigating workload on different shift-phases and between work-shifts, with two custom-made tools. The first one enabled workload assessment by manipulating intrinsic load (task difficulty) and extraneous load (time pressure) in a working-memory task. The second tool was a questionnaire based on the theoretical concepts of work-demands, control, and psychosocial support. Two additional dimensions suspected to contribute to job-perception, i.e., work–family conflicts and availability of human and technical resources were also explored. Results of workload assessments were discussed in light of operators’ alertness and job-performance.

## INTRODUCTION

Two fundamental research questions have driven working memory research during the past four decades. The first concerns the role of attention during information processing in working memory. It will be outlined briefly in that it is closely related to the second research topic on which the present contribution focusses, i.e., the factors that determine the limitations of working memory, and thus workload. Workload is a closely related, partially or totally overlapping concept of cognitive load. A precise definition is elusive, but a commonly accepted definition of workload has been proposed by Hart and Staveland (1988): the perceived relationship between the amount of mental processing capability or resources and the amount required by the task.

The purpose of the present contribution is to provide a comprehensive overview of workload theories and assessment of workload, more especially in the work place. In the literature, workload has been addressed in different, but complementary, ways in the fields of ergonomics and of occupational psychology. We will review more especially cognitive load theory that proposes a distinction between different load categories and factors in working memory, and job-strain models that consider job-demands in relation to other job-related dimensions. Both these approaches will be discussed together with an additional concept that is central to those studies focusing on workload in shift-work or night-work conditions, i.e., alertness variations and thus performance variations across the 24 h-day in shift-workers. Concepts and considerations derived from these theoretical approaches enabled the development of a multi-disciplinary approach of workload that will be described in a later section. A final section will report applications of this original approach in field studies in relation to alertness and job-activity.

### SINGLE VERSUS MULTIPLE MENTAL RESOURCES DURING INFORMATION PROCESSING

One of the first models proposed to account for cognitive performance by mental resources can be found in Kahneman’ (1973) influential book on attention. In this model, human performance is supported by a general pool of mental “effort” and the demand of task for these limited resources is emphasized. When a subject engages in a demanding task, this single multipurpose pool of resources saturates, leaving less room for additional processing regardless of the task domain, and performance breaks down.

In contrast, Wickens’ (1984) multiple resource theory identified attentional resources that are separate from one another along four dimensions: the stage of processing (perceptual and working memory tasks versus selection and execution of action), the type of processing code in perception, working memory (Baddeley, 1986) and action (spatial activity versus verbal activity), and the modalities of input and output (auditory versus visual). The fourth dimension was introduced in a later development, by distinguishing within visual channels focal vision and ambient vision (Wickens, 2008). According to this theory, the human operator has several different pools of resources that operate independently and that can be tapped simultaneously. Excess workload would arise by a task using the same resource and may then result in errors or slower task performance.

A more integrated view proposes that late/central processing, for example high working memory load in a visual–verbal task, interacts with early/sensory processing, for instance of irrelevant sound (see for instance, Sörqvist et al., 2012). This interpretation may account for instance for higher recall of auditory rather than visually presented verbal material, as a result of a longer-lasting acoustic-sensory trace and/or higher temporal distinctiveness of heard lists of items (Galy et al., 2008, 2010; Mélan and Galy, 2012).

Both single and multiple resource models posit that if task demand exceeds capacity of resources, performance breaks down. They propose that attentional resources would protect the limited processing space of working memory from overload. In this respect, the concept of mental resources has a significant contribution to the understanding of workload. The reader may refer to other sections in this volume for further discussion of the main components of attention, in particular intensity, selectivity, and control of the underlying processes. The purpose of the present contribution is a better understanding of workload in real-job situations rather than of the various components of mental resources or attention. The next section thus focuses on the factors that determine workload in the work place.

### COGNITIVE LOAD FACTORS AND CATEGORIES IN WORKING MEMORY

In his nominal paper of Miller (1956) was the first to suggest that working memory capacity was limited to a defined number of digits of information. A central issue of that and subsequent theories was to describe how people might organize information in a capacity-limited and time-limited short-term memory store, for instance by chunking or by schema construction. Such processes would depend on cognitive load, defined by the mental activity imposed on working memory, or by the load related to the executive control of working memory. An important issue was to define the factors that determine cognitive load in regard with its time-limitations and/or its capacity-limitations. Time-limitations of working-memory have been proposed for instance by the time-based resource-sharing model. According to this model, cognitive load depends on the proportion of time during which a given activity captures attention in such a way that the refreshment of memory traces or any other activity that requires attention is impeded (Barrouillet et al., 2004). Other models, focusing on the capacity-limitations of the working memory store, define cognitive load as the total amount of mental activity imposed on working memory. One of these models, known as cognitive load theory, emphasizes the capacity limitations of working memory on learning during instruction (Sweller et al., 1998).

Cognitive load theory distinguishes three different cognitive load categories. Intrinsic cognitive load, referring to the number of cognitive units to be maintained and processed in working memory while performing a task, is due to the intrinsic nature (difficulty) of to-be-learned information. Extraneous cognitive load refers to cognitive and non-cognitive components of the environment that contribute to the manner in which information is presented (instructional materials, time pressure, noise …). Germane load results from the processing, construction and automation of schemas. For complex problem-solving tasks, requiring a relatively large amount of cognitive processing capacity, only a limited capacity may be devoted to schema construction (Sweller, 2006). Extraneous cognitive load may, however, be reduced by instructional design for instance, thereby increasing the amount of resources available to process intrinsic load and germane load. This theory provides a general framework to control the conditions of learning in order to “redirect learners’ attention to cognitive processes that are directly relevant to the construction of schemas” (Sweller et al., 1998, p. 249).

Recently, cognitive load theory has been implemented in the field of ergonomics, by exploring various cognitive load measures in a mental arithmetic task, typically involving working memory (Galy et al., 2012; Galy and Mélan, 2013). The study revealed additive effects of intrinsic load (high task difficulty) and extraneous load (high time pressure) on working memory performance and on mental efficiency. Mental efficiency has been defined by Paas and Van Merriënboer (1993) and combines an objective workload measure (performance) and a subjective workload measure (mental effort). The study also showed that the combined disruptive effects of intrinsic and extraneous load factors were further enhanced when subjects’ alertness was low, i.e., in the morning (Thayer, 1989). No such effect of alertness was observed when either extrinsic or intrinsic load was high while the other load factor was kept at a low level (low task difficulty or low time pressure). Further, alertness affected not only mental efficiency and performance but also a psycho-physiological measure of workload (i.e., differential heart rate), indicating the robustness of this effect. The authors suggested that decreased alertness observed in the morning would result in more limited cognitive resources. The latter would be entirely allocated to deal with the more basic intrinsic and external cognitive load factors, leaving only limited resources to elaborate efficient strategies and thus for germane load. Conversely, in the afternoon, when alertness was high, more cognitive resources could be allocated to working memory, thereby enabling the generation of efficient strategies despite high intrinsic and extraneous loads.

**FIGURE 1:**
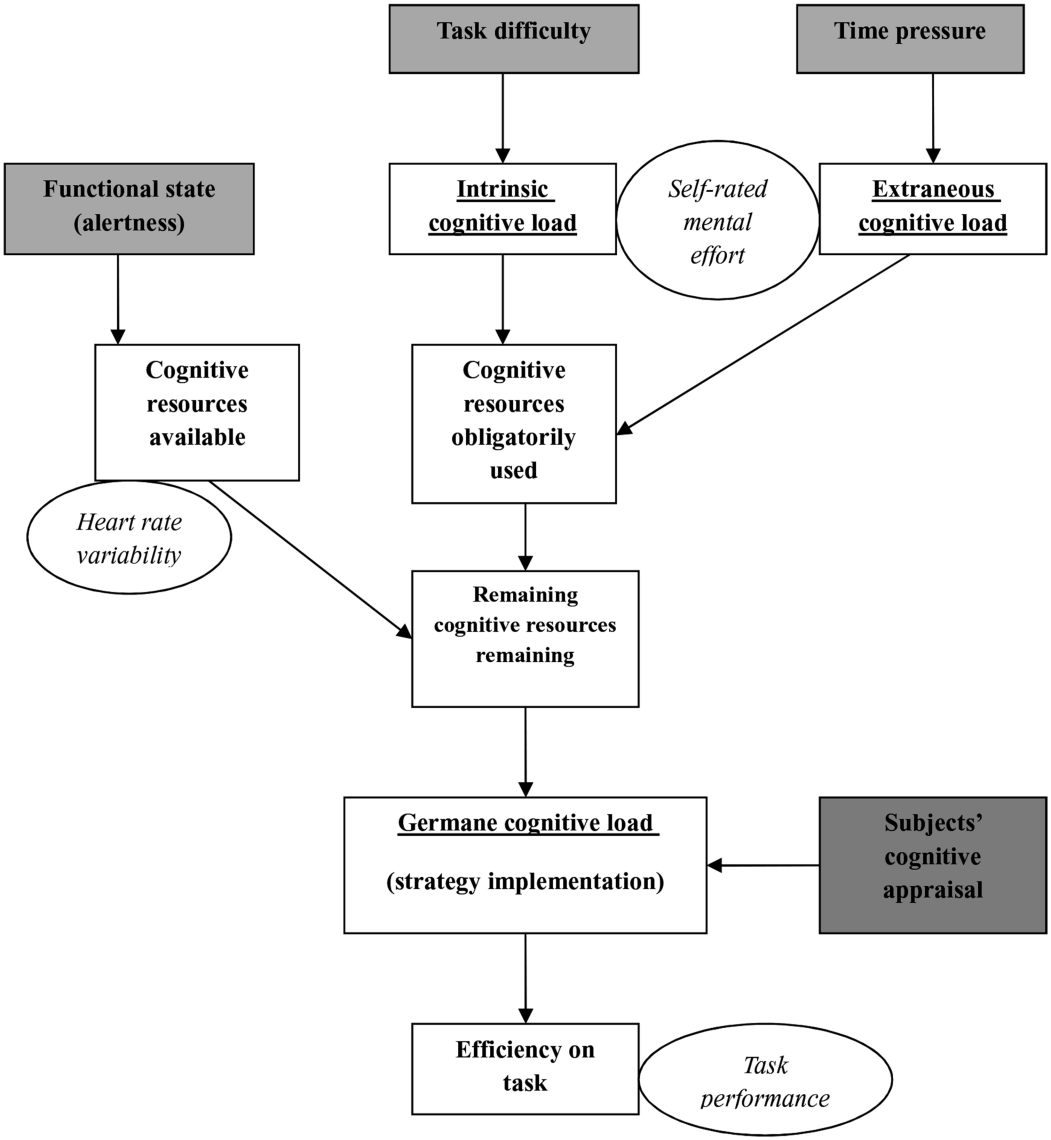
**Graphical representation of putative relationships between cognitive load factors and cognitive load categories in working memory tasks (Galy et al., 2012)**.

In line with this interpretation, alertness has been reported to be closely related to an individual’s body temperature and its diurnal variations that are generally considered to reflect his/her functional state over the 24 h-day (Cariou et al., 2008). Accordingly, the authors proposed a modified cognitive load model and introduced alertness as a marker of the resources that are available for germane load (**Figure 1**). The model shows how the effects of well-defined load factors may be modulated by alertness variations. This study thus raised interesting perspectives concerning workload investigations in the work place, and more especially in those job-situations involving continuous work over the 24 h-day.

### ALERTNESS AND COGNITIVE PERFORMANCE IN THE WORK PLACE

The shift-work literature provides clear evidence of alertness variations across the 24 h-day in the work place, and of correlative performance variations in neuropsychological tasks. Shift-workers’ alertness trend recorded in the work place is comparable to the one reported in controlled laboratory conditions, i.e., an increasing trend across the day, reaching its maximum in the afternoon, decreasing thereafter, first slowly, then steadily to reach its minimum between 02:00 and 06:00 (Cariou et al., 2008). Hence, shift-workers’ self-rated alertness variations across the 24 h-day have been shown to be correlated with their performance in mnemonic and discriminatory tasks. Further, decreased alertness during the night-shift affected performance in these tasks, but only in the most difficult task conditions (Galy et al., 2008), like reported above in the mental arithmetic task (Galy et al., 2012). Likewise, immediate recall of verbal material was lowest when alertness was also lowest, but only in the cognitively more demanding task conditions, i.e., for recall of visually rather than of auditory presented word-lists, and of long rather than of short word lists (Galy et al., 2010).

Higher workload on the first shift-hour compared to the remaining time on shift has also been proposed to account for enhanced job-performance (Andorre and Quéinnec, 1998), but also higher heart rate and self-rated tension during supervisory control of a dynamic system (Cariou et al., 2008). In such job-situations cognitive load would be particularly high on the beginning of each shift, including on the night-shift, as on this shift-phase operators are involved in the built-up of situation awareness and of a mental representation of the system’s state and the programming of the operations to be performed on the remaining time of the shift. The main objective of assessing and predicting workload in work settings is to achieve evenly distributed, manageable workload and to avoid overload or underload, arising for instance while performing monotonous tasks over time and/or by a lack of stimulation (Dunn and Williamson, 2012). Both mental underload and overload have been shown to be associated with higher incident and accident rates (Flatley et al., 2004; Chiron et al., 2008).

The effects of under- and overload would be most prominent during the night, as indicated by a higher probability of an operator being involved in an accident or injuring himself at times when he/she would normally be asleep (Folkard and Tucker, 2003). More generally, “being exposed to the circadian low, extended time awake, or reduced duration of sleep will impair performance” (Akerstedt et al., 2004; Akerstedt, 2007, p. 209). As these situations are typically associated with shift-work, theoretical models of the sleep and circadian system have been proposed to predict fatigue and/or alertness and, by inference, fatigue-related errors, and accidents/incidents, essential for the development of fatigue risk management systems in safety-related job-situations (Folkard and Lombardi, 2006; Dawson, 2012).

Findings of the shift-work literature then suggest that specific characteristics of job-situations, including shift- and night-work, may be regarded as environmental components that potentially enhance extraneous cognitive load in operators while performing their job activity. These components probably vary between job-situations as has been suggested by Siegrist (2010), and should be considered together with task-specific components (intrinsic load) in order to determine workload. This review of the literature thus further favors the idea that alertness may be viewed as an indicator of the cognitive resources available to generate efficient strategies in a task in light of the different load factors (Galy et al., 2012).

### WORK DEMANDS, FATIGUE, AND PERFORMANCE IN THE WORK PLACE

An individual’s perception of his/her work environment (i.e., psychosocial features of the work environment) influences safety and performance on the work place and, at a long run, his/her mental and physical health (Costa, 1996). These relationships have been commonly addressed in the demand–control–support model (Karasek, 1979; Theorell and Karasek, 1996). According to this model, a combination of high task demands (psychological) and low control predicts job strain, and this more especially when social support is low. Several studies reported for example that work stressors, including autonomy and demand, are related to the frequency of occupational injuries and near-misses (Hemingway and Smith, 1999; Goldenhar et al., 2003). Parker et al. (2001) found that these factors influenced the self-reported level of safe working. A mismatch between work demands and the resources available to meet them (i.e., control and social support) would indicate that employees are focused on managing workload or that they are experiencing some level of strain which makes them prone to errors in their work as the result of a performance decrement (Phipps et al., 2012). An alternative model, the effort–reward imbalance model, posits the interplay between job-related psychological effort and reward, and individual differences in the level of commitment to work, as predictors of strain (Siegrist, 1996; Siegrist et al., 2004). Several studies showed that both models contribute to the prediction of safety performance and safety climate ratings (Phipps et al., 2012).

In a different approach, the work–family conflict model proposes that strain arises when “participation in one role (work role or other life roles) makes it difficult to fulfill requirements of another” (Greenhaus and Beutell, 1985, p. 76). In a society that operates around a 09:00–17:00 work schedule, employees working in the early morning, during the evening or during the night are more readily exposed to conflict roles between these two major areas of life. The disrupting “effects of shift work on performance efficiency, accidents, and family and social life” have been described as early as by Rutenfranz et al. (1977). More recent studies showed that shift-work, but also work-related demands, and job insecurity, figure among the risk factors for the onset of work–family conflict, whereas decision latitude and social support (co-worker and supervisor) protect against work–family conflict (Jansen et al., 2003).

While the demand–control–support model and the effort–reward imbalance model have received much attention in the literature, studies focusing on shift-work highlight more especially the work–family conflict model. The work–family imbalance model provides a link between the short-term effects of shift-work on fatigue, and its long-term effects on general health and well-being. It posits that individuals usually try to achieve a balance between work and family requirements and that meeting demands of both areas frequently results into sleep loss in employees working evening- or night-shifts (Caldwell, 1997). The fatigue associated with sleep loss, shift work, and long duty cycles for instance, can cause him/her to become inattentive, and inefficient. Experimental studies clearly demonstrated that short-term sleep deprivation results in alertness and cognitive performance decrements correlatively to a decrease in brain activity and this more especially in those brain regions mediating attention and higher-order cognitive processes (Thomas et al., 2000). Accordingly, in safety-related job-situations fatigue may constitute an insidious threat because of alertness and performance impairments and the insecurity it may generate. At the long run, an imbalance between employment requirements and family responsibilities may result in a disruption of physical, mental, and/or social well-being (Roth and Moore, 2009). High levels of fatigue, need for recovery, poor sleep quality, poor general health, work–family conflict, and insufficient leisure time were thus reported to be associated with an increased risk of leaving shift-work (van Amelsvoort et al., 2004).

These and other findings of the job-strain literature that may not be summarized here, provide clear evidence indicating that the consequences of a stressful work environment depend on “many ‘intervening variables’ concerning both individual factors (e.g., age, personality traits, physiological characteristics), as well as working situations (e.g., workloads, shift schedules) and social conditions (e.g., number and age of children, housing, commuting)” (Costa, 1996, p. 9).

### A MULTI-DISCIPLINARY APPROACH OF WORKLOAD VARIATIONS WITHIN A WORK-SHIFT AND ACROSS WORK-SHIFTS

Despite the difficulties of finding a precise definition of workload, a number of tools have been proposed to operationalize these theoretical concepts. Mental workload may be evaluated by recording of psychophysiological components, observing overt task performance, or rating subjective tools. In the work place, mental workload is mostly assessed by subjective self-rating scales and questionnaires, like the NASA-task load index (NASA-TLX; Hart and Staveland, 1988) and the subjective workload assessment technique (SWAT; Reid and Nygren, 1988). The NASA-TLX, for instance, is a multi-dimensional rating procedure that derives an overall workload score based on average of ratings on six subscales including mental, physical, and temporal demands. It allows subjective workload assessments on operators working with various human–machine systems.

A broader description of workload may be obtained by exploring an individual’s perception of his/her work environment with self-rating questionnaires. These tools may be used in any job-situation, whether they involve or not a human–machine system. The main interest of this approach arises from the fact that workload or work-demands are highlighted in relation to other perceived job-features, i.e., the resources available on the work place to meet the demands in the demand–control model (Theorell and Karasek, 1996), reward and work commitment in the effort–reward model (Siegrist, 1996), and putative conflicts with family roles in the work–family imbalance model (Greenhaus and Beutell, 1985). As indicated above, investigating work demands together with other job-features enables determining whether a work environment is stressful or not. A detailed description of operators’ workload may then be achieved by combining, in the work place, the assessment of employees’ perception of their work environment with an experimental investigation of various workload measures in response to controlled manipulations of load factors.

The questionnaires used to specifically test either of the job strain models proved indeed to be useful to explore psychosocial job characteristics in a variety of job-situations (Karasek et al., 1998). There remain, however, some theoretical and practical questions to be addressed in this research field. For instance, the subjects’ responses are most probably based on the job-experience they gained over a rather long time period, though the time-frame they should consider is generally not specified. This may then lead to biased assessments. It also remains unclear how a subject’s overall perception of his/her work environment, and in consequence a demand–control (or resource) mismatch or a work–family imbalance, build up over time. In other words, it may be interesting to determine whether the contribution of a given dimension (and of its sub-dimensions) is constant over time, and what specific job characteristics may modify the contribution of each dimension. It may for instance be the case that an employee’s perception of his/her work environment depends, in addition to overall work organization (work schedule), also on more focal aspects, including work-shift, staff on shift (i.e., reduced staff on the night shift for instance), hours on shift, and beginning of the morning shift. This idea is favored by the fact that these work organization features have been shown to affect employees’ performance, as outlined above. Moreover, shifts starting before 06:00 have been reported to be associated with higher levels of circulating cortisol (stress-related hormone; Bostock and Steptoe, 2013), and decreased alertness in the late morning (Tucker et al., 1998) compared to late shifts. A backward rotation schedule was reported to be related to an increased need for recovery and poor general health, when compared with a forward rotation schedule (van Amelsvoort et al., 2004).

It may furthermore be argued that job-perceptions may vary within a given shift, as aforementioned for other psychological measures (i.e., alertness and performance) which were shown to vary according to morning-shift beginning and shift-duration (Tucker et al., 1998), time on shift (Mélan et al., 2007), high workload on shift-beginning (Andorre and Quéinnec, 1998; Cariou et al., 2008), and task load (Galy et al., 2008). It is tempting to speculate that if in a work environment workload is objectively enhanced on shift-beginning for instance, then this specific feature would also be uncovered by employees’ self-reports. Even more, a self-rating tool would enable investigating on different phases of a given shift and across shifts this feature along with other dimensions of the work environment.

In light of these considerations, we developed tools and a specific methodology in order to investigate workload in the work place, both in the context of job perception and in a controlled experimental design. On one hand, we designed a self-rating questionnaire referring to the theoretical concepts of the demand–control model (job demand, control and social support), completed by two other relevant concepts, i.e., work–family conflicts and availability of technical and human resources. We argued that the availability of technical and human resources may contribute to meet job demands more accurately, together with the well-documented resources already mentioned. In addition, control and its sub-dimensions (autonomy and skill expression) may critically depend on the availability of technical and human resources. Data summarized in the previous section further provided clear evidence of an incidence of work–family conflicts on sleep and fatigue, and thus on job-perception on a given shift or a given shift-phase.

In consequence, our job perception questionnaire addressed work demands (psychological, 19 items; physical, six items), control (autonomy, four items; skill expression, eight items), social support (supervisor, five items; co-worker, three items), work–family conflicts (five items), technical and human resource availability (five items). Subjects rated all 56 items on a six-point Lickert-type scale and a mean score was calculated for each dimension (a high mean score indicating high demands, control, etc.). Work demands would enable assessing workload. Cronbach’s alpha’s indicated a high inter-item reliability for each dimension (in each case >0.800).

We also designed an experimental procedure in order to investigate in detail workload, and its variations according to intrinsic and extraneous load. Among the load factors of interest in a work environment, and that can be implemented in an experimental design, task difficulty and time pressure appeared to be most relevant. Besides task-difficulty or -complexity, time pressure has indeed proved to be one of the most common stressors in the work environment, where time may be part of a mediating process that influences perception of control (Koslowsky et al., 1995). The experimental procedure enabled testing the effects of the two load factors separately and simultaneously in a working memory task by recording performance measures and subjective load measures. More especially, in a mental arithmetic task each of 32 trials started with the presentation of a two- or three-digit number on a computer screen. Subjects had to add “5” and “18” to the displayed digit respectively in the low difficulty and high difficulty conditions, either without time-pressure or under time-pressure (respond within 8000 ms). Subjects thus performed four experimental conditions: low difficulty and low time-pressure, low difficulty and high time-pressure, high difficulty and low time-pressure, high difficulty and high time-pressure (Galy et al., 2012).

In the field studies described in the next sections, the self-rating job-perception questionnaire and the experimental investigation of load factors were combined to provide a detailed description of workload in the work place. Given that workload has also been shown to depend in a complex manner on several personal, situation-related, and task-related factors including sleep loss, and job characteristics, which in turn affect alertness and (safety) performance (i.e., Costa, 1996), the studies also included alertness and real-job activity measures.

## EXPERIMENT 1: WORKLOAD, ALERTNESS, AND JOB-PERCEPTION ARE RELATED TO WORK-ACTIVITY

The aim of a first experiment was to explore whether cognitive load measures, job-perception (including work demands), and alertness vary according to on-shift time in air traffic controllers, and to explore whether workload measures were associated with operators’ general state (alertness) and with their job-activity.

Nine out of the eleven controllers of the French Air Force working in a test flight control center, volunteered to participate in the study. They were aged between 34 and 56 (mean 42.7), had a 10 year work experience, and worked on week-days, starting at 08:30 or 10:00. Controllers were in charge of individual test flights of to-be-commercialized aircrafts. They had to make sure that aviators had the possibility to test the proper operation of flight instruments in a sufficient air space and time-frame, despite commercial aircrafts arriving or leaving the regional airport, en-route flights on regular air routes and domestic flights. Test flights lasted a mean of 20 min. The first flight was scheduled approximately 1 h after work-beginning, and subsequent flights were scheduled on an irregular base. Two activity categories were recorded: “communications” and “radar activities” (other overt behaviors were finally discarded as they represented less than 5% of the events).

Workload was investigated by using the tools described in the previous section, i.e., in a working memory task by manipulating intrinsic and extraneous task load separately and simultaneously, and directly in the work context by rating the different dimensions of the job-perception questionnaire (work demands, control, social support at work, work–family conflicts, and availability of technical and human resources). Alertness and perceived tension were determined using Thayer’s adjective check-list. Briefly, controllers rated either of four responses [“I feel very …,” “I feel a little …,” “I don’t know,” or “I don’t feel ...” scoring respectively 4, 3, 2, and 1 point(s)] for each of 20 adjectives relating either to alertness or tension. Workload measures and alertness were collected three times: 1 h after shift-beginning, in the middle of the shift, 1 h prior shift-end. In consequence, control activities were only recorded during the first test flight as it was operated within the first hour of the shift and the data could thus be confronted to the other measures recorded 1 h after shift-beginning. The irregular flight schedule of later flights did not fit with the procedure described above, so that no further control activity recordings were performed (Maruque et al., 2013). As several variables did not meet the criteria of a normal distribution, non-parametric comparisons were performed for each measure across the three shift-phases by using Friedman’s test (three related samples with nine observations) and *post hoc* pair-wise comparisons with Wilcoxon’s test. Significant associations between variables were tested by using Spearman’s correlation test. Results are presented successively for each of the tools used, before investigating correlations between alertness, cognitive performance and job perception and of each measure with real job-activity (on shift-beginning).

**FIGURE 2:**
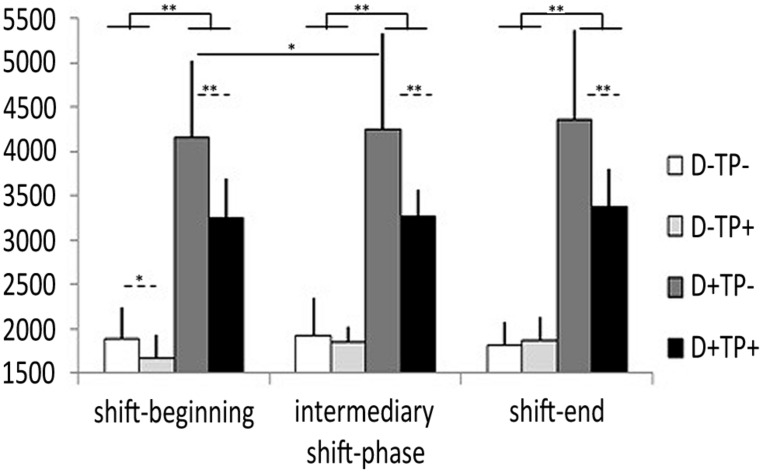
**ATCs’ response latencies (in ms; mean ± SD) in four conditions of a mental arithmetic task characterized by high versus low difficulty (D) and time-pressure (TP).** ^∗^*p* < 0.05 and ^∗∗^*p* < 0.01.

**FIGURE 3:**
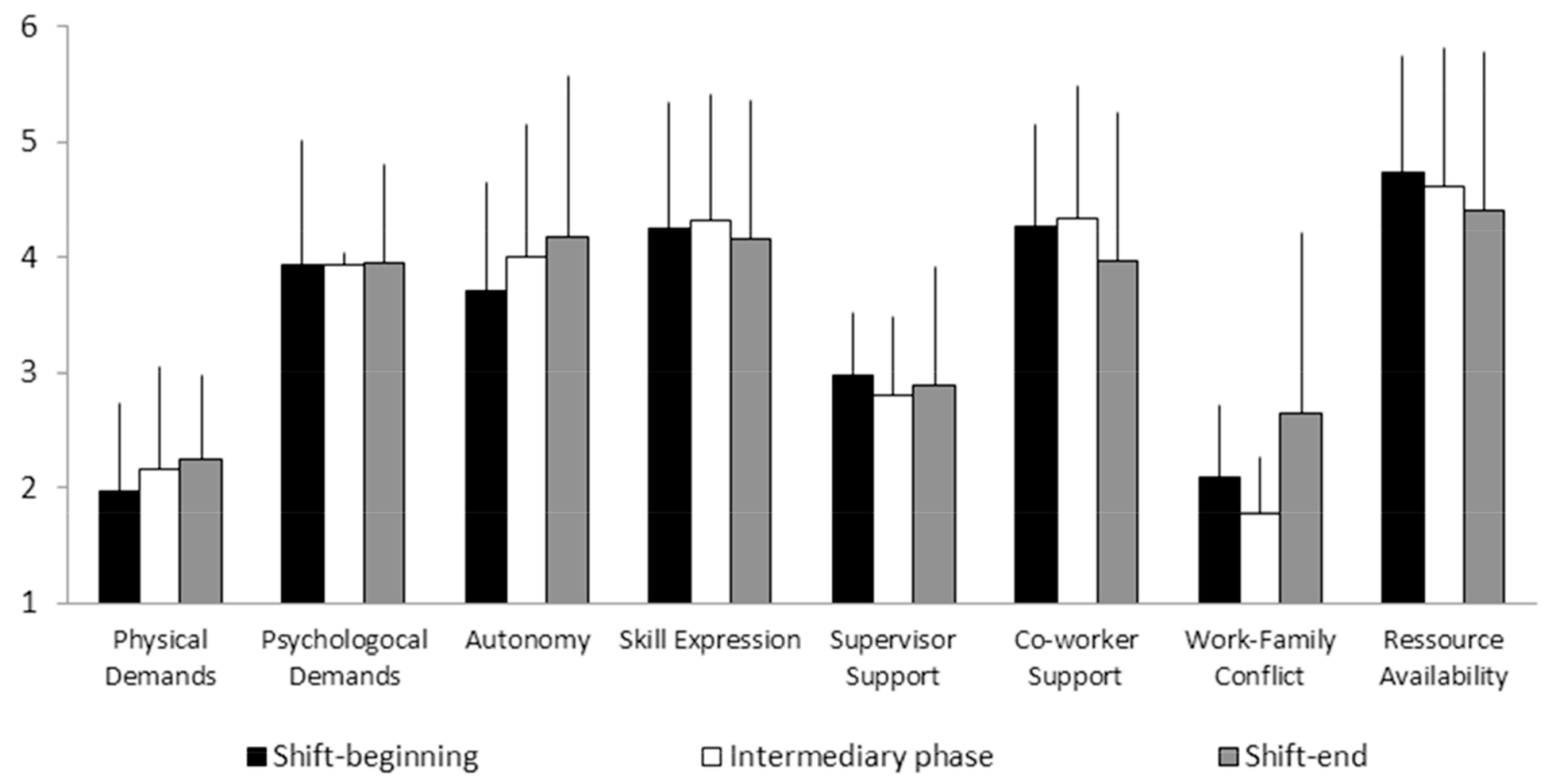
**Job characteristics (mean ± SD) perceived by ATCs on three shift-phases**.

– **Figure 2** illustrates working memory performance expressed by response latencies so that low response latencies indicate high performance and vice versa. Friedman’s test was used to compare task performance between the four task conditions separately on each shift-phase, and for each task condition across the three shift-phases. Results indicated significant performance differences according to task condition on shift-beginning (*W* = 25.13, *p* < 0.001), shift-middle (*W* = 24.6, *p* < 0.001), and shift-end (*W* = 24.6, *p* < 0.001). *Post hoc* tests indicated higher working memory performance (in each case, *p* < 0.008) when intrinsic cognitive load was low (task difficulty, labeled D- in the figure) rather than high (labeled D+). When task difficulty was high, performance was higher with high time pressure (D+TP+) rather than with low time pressure (D+TP-; in each case, *p* < 0.008). When task difficulty was low, a similar effect of high time-pressure was observed but only on shift-beginning (*p* < 0.021) while no such effect occurred on the middle and end of shift. This result then indicates a performance decrement across the shift with increasing extraneous load.

– Correlatively, air traffic controllers’ self-rated alertness decreased between the beginning (*M* = 1.56, SD = 0.91) and end of shift (*M* =1.07, SD = 0.90), while their self-rated tension remained low throughout the shift (between 0.28 and 0.33). Friedman’s test did not reveal significant alertness or tension variations across the three shift-phases, though pair-wise comparisons indicated that ATCs reported significant higher alertness on shift-beginning than on shift-end (*Z*= 1.96, *p* < 0.050).
– As indicated by **Figure 3**, ATCs’ perception of the different dimensions of their work environment appeared to remain stable across the shift. This impression was confirmed by statistical analysis revealing no significant differences between shift-phases for the perception of either job dimension. However, Spearman’s correlation tests performed separately on each shift-phase revealed significant associations between the different job dimensions. On shift-beginning, psychological demands were positively correlated with job control (autonomy: ρ = 0.840, *p* < 0.005; skill expression: ρ = 0.917, *p* < 0.001). Further, demands, control, and co-worker support were positively correlated with human and technical resource availability (psychological demands: ρ = 0.828, *p* < 0.006; autonomy: ρ = 0.717, *p* < 0.030; skill expression: ρ = 0.870, *p* < 0.002; co-worker support: ρ = 0.681, *p* < 0.043). Technical and human resource availability was also associated with co-worker support on the remaining shift-phases (middle of shift, ρ = 0.953, *p* < 10^-4^; shift-end, ρ = 0.734, *p* < 0.02), and with psychological demands on shift-end (ρ = 0.667, *p* < 0.050).

– Correlation analyses between the different measures and real-job activity on shift-beginning revealed significant associations of total flight control activities with working memory performance (negative correlations with response latency) when task difficulty was high (D+TP-, ρ = -0.703, *p* < 0.04), and when time pressure was high (D-TP+, ρ = -0.783, *p* < 0.013). A similar relationship between control activities and alertness fell short of significance (*p* < 0.09), indicating nevertheless that control activities tended to be highest when alertness and tension were also highest, and vice versa. High perceived supervisor support and low self-rated tension were also associated with high working memory performance when intrinsic load was high (respectively, ρ = 0.-667, *p* < 0.050; ρ = 0.720, *p* < 0.029). The rate of communicative events was however negatively correlated with perceived supervisor support (ρ = 0.898, *p* < 0.001), indicating that ATCs perceived low support when flight control involved a high rate of communicative items.

In summary, the findings of high alertness and cognitive performance on shift-beginning, together with the fact that a test flight was systematically scheduled shortly after shift-beginning in the test flight control center, may indicate that controllers anticipated high workload on shift-beginning in this particular job-situation. A similar interpretation has been proposed in previous studies demonstrating significant higher job-performance, perceived tension, and heart rate 1 h after shift-beginning compared to the remaining shift-time in situations involving supervisory control of a dynamic (Andorre and Quéinnec, 1998; Cariou et al., 2008). In favor of this interpretation, positive relationships were observed on shift-beginning between cognitive performance, job-activity, and alertness. In addition, job demands and various job resources were rated at a high level and significantly correlated to each other on this shift-phase.

According to Karasek’s model (Theorell and Karasek, 1996), high job demands associated with high control would indicate that ATCs perceived their work situation as an “active job situation” or a “passive job situation,” but not as a “high strain” job involving, on contrary, high job demands and low job control. Psychological demands and control were not significantly associated with co-worker support unlike the model’s prediction. However, the three main dimensions (i.e., job demands, control and social support) were associated with an additional resource considered in the study, i.e., availability of human and technical resources. It is thus tempting to speculate that adequate availability of these resources may have accounted for ATCs’ perception of an active/passive job-situation. More especially, when this kind of resource is available in the work environment it may enable more efficient control, thereby providing a better match of high job demands.

This interpretation was favored by the findings that psychological demands, and technical and human resource availability were rated at a high level throughout the shift, and that both measures were correlated on shift-beginning and on shift-end. Co-worker support was also rated at a high level throughout the shift and significantly associated with technical and human resource availability. These finding are important as social support has been repeatedly reported to be associated with safety compliance and behavior (Lin et al., 2012; Li et al., 2013), that are essential for ATC.

On the other hand, job-perception did not notably vary between shift-phases and work–family interferences were low on contrary to our predictions. In order to further establish the interests of the methodology developed in this study and the relevance of considering additional resources in job perception research, we performed a second study in operators also working in the field of aeronautics (satellite control) but according to a three-shift system. For the reasons outlined above, variations in workload (objective or subjective) are indeed most likely to occur between shifts even though they would not necessarily occur within the same shift.

## EXPERIMENT 2: WITHIN- AND BETWEEN-SHIFT VARIATIONS OF WORKLOAD, JOB-PERCEPTION, AND ALERTNESS

A second study aimed to test several issues raised by the previous study. First, it was important to establish whether the reported results (alertness and performance decreases across the shift) were specific to the job-situation under investigation or whether they could be generalized to other job-situations involving high workload on shift-beginning, as has been reported for other load measures during supervision of a dynamic process (Andorre and Quéinnec, 1998; Cariou et al., 2008). Second, it was not clear from the previous study why job-perception remained stable throughout the shift in contrast to significant changes across the shift of other psychological measures. If the observed alertness and performance decreases were the result of well-documented factors including high workload on shift-beginning, prior sleep loss (Caldwell, 1997; Galy et al., 2010, 2012; Galy and Mélan, 2013), or on-shift time (Tucker et al., 1996, 1998; Mélan et al., 2007), job-perception might have been expected to vary in a similar way. Third, operators were on duty only during the day in the test flight control center, so that it was still not clear whether and to what extent job-perception varies between work-shifts as a function for instance of staff, work organization, or circadian influences (Rutenfranz et al., 1977; Folkard and Tucker, 2003; Galy et al., 2008; Mélan and Galy, 2012).

Operators (*n* = 8) participating in the second field study were satellite controllers, aged between 28 and 59 (mean age 45.7). They worked three shifts: a morning-shift starting on 07:00 and ending on 12:00 or 16:00, an afternoon-shift starting either on 12:00 or 16:00 and ending on 21:00 and a night-shift (21:00–07:00). The same procedure and methodology were used than in the previous experiment. Briefly, three recordings were performed on each shift (1 h following shift-beginning, middle of the shift and 1 h prior shift-end), except for the 5 h-day-shifts where only two recordings were performed. Due to the particular shift-scheduling system, we compared the data collected during the night-shift to those recorded on the day-shifts (pooling data from the morning and afternoon-shifts). Workload was assessed on three (two on short day-shifts) 1-h periods by a job activity index corresponding to the sum of activities performed (phone calls, alarms, supervisory activity, archiving activity), and by the job-perception questionnaire. On each recording they also completed Thayer’s adjective check-list, and performed the working memory task in the conditions of low intrinsic load (low difficulty) associated with either low or high extraneous load (high or low time pressure), described in experiment 1. Cognitive load in the experimental task was assessed by objective measures (response latency, number of correct responses) and by subjective measures (mental effort, perceived task difficulty, time pressure, and task commitment). For the latter, participants were asked to rate 10 cm visual analog scales following completion of each task condition.

Skewness and kurtosis tests indicated a normal distribution of the data for most variables, except for tension ratings and for job activity which were therefore discarded from the parametric analyses. Analyses of variance with two repeated measures investigated the effects of shift (day-shift versus night-shift), and of shift-phase (shift-beginning, shift-middle and shift-end), and more especially interactions between the two factors. Correlation analyses with Pearson’s test explored the putative relationships between work activity (real-job activity and job-perception) cognitive performance and alertness, separately on each shift-phase of the day- and night-shifts.

– Analysis of Thayer’s questionnaire indicated significant higher alertness on day-shifts (*M* = 2.56, SD = 0.37) compared to night-shifts [*M* = 2.04, SD = 0.47; *F*(1,6) = 7.29, *p* < 0.04]. A significant quadratic trend of shift-phase [*F*(1,6) = 6.00, *p* < 0.05] was also observed. **Table 1** shows that alertness was higher on shift-beginning compared to the two remaining recordings of the shift, but *post hoc* comparisons were not significant. No interaction occurred between the two factors.

**Table 1:** Operators’ alertness, working memory performance, and job activity on each shift-phase.

	Measure	Shift-beginning *M* (SD)	Intermediary shift-phase *M* (SD)	Shift-end *M* (SD)	*p*
Thayer’s check-list	Alertness index	2.71 (0.39)	1.99 (0.45)	2.19 (0.40)	0.05
Working memory task	Response latency (ms)	2585 (0.338)	2344 (0.247)	2264 (0.271)	0.03
	Perceived difficulty	7.07 (0.62)	6.09 (0.80)	5.71 (0.55)	0.04
	Perceived effort	4.81 (0.67)	5.35 (0.88)	5.43 (0.73)	0.01
Job activity:		
– Day-shifts	Activity index	1.71 (2.61)	3.83 (3.06)	1.57 (1.53)	0.05*
– Night-shifts	Activity index	1.67 (1.50)	0.50 (0.54)	3.66 (7.55)	NS
Job perception questionnaire	Physical demands	1.84 (0.15)	1.96 (0.13)	2.06 (0.20)	NS
	Psychological demands	2.05 (0.23)	1.87 (0.17)	1.74 (0.12)	NS
	Autonomy	3.65 (0.27)	3.20 (0.33)	3.27 (0.30)	NS
	Skill discretion	3.02 (0.21)	3.00 (0.20)	2.92 (0.16)	NS
	Supervisor support	1.57 (0.22)	1.39 (0.11)	1.50 (0.18)	NS
	Co-worker support	2.10 (0.21)	1.64 (0.21)	1.65 (0.15)	0.06
	Work–family conflict	1.54 (0.32)	1.48 (0.32)	1.52 (0.33)	NS
	Resource availability	3.30 (0.21)	3.15 (0.29)	3.04 (0.31)	NS

For each variable the mean (*M*) and standard deviation (SD) are indicated, followed by the *p*-value of the ANOVA (*non-parametric comparison).

– Analysis of working memory performance revealed neither an effect of shift, nor an interaction between shift and phase of shift. However a significant effect of shift-phase occurred for response latency when both intrinsic and extraneous load were low [*F*(1,6) = 8.94; *p* < 0.03]. Though *post hoc* tests were not significant, **Table 1** indicates decreasing response latencies and thus increasing task performance across the shift. Operators’ perceived task difficulty [*F*(1,6) = 6.41, *p* < 0.04] and mental effort [*F*(1,6) = 13.65; *p* < 0.01] also varied across the shift. *Post hoc* tests indicated a significant higher perceived effort on shift-end compared to shift-beginning (*p* < 0.03), and a decreasing but non-significant trend for perceived difficulty. No significant effect or interaction was observed for the number of correct responses.
– Real-job activity showed important inter-individual variations as expressed by high standard deviations (**Table 1**), in particular on the end of the night-shift where an alarm was triggered repeatedly. Non-parametric analysis with Friedman’s test revealed nonetheless significant differences across day-shifts (*W* = 6.99, *p* < 0.05), with significant higher activity levels on the middle than on the beginning of these shifts (*post hoc* Wilcoxon test: *Z* = 2.20, *p* < 0.03). Comparisons on each shift-phase further revealed a significant higher activity index on the middle of the day-shifts compared to the night-shift (*Z* = 2.21, *p* < 0.03).
– As shown by **Table 1**, job perception remained fairly stable across shift-phases. Overall, resources (autonomy, skill discretion, resource availability) were rated at a higher level than job-demands (physical and psychological demands, work–family interferences). Statistical analyses revealed a main effect of shift for all the dimensions investigated except for work–family interferences (**Table 2**). Thus, psychological demands [*F*(1,6) = 5.16, *p* < 0.06], skill discretion [*F*(1,6) = 31.94, *p* < 0.001], social support [supervisor *F*(1,6) = 9.75, *p* < 0.02; co-worker *F*(1,6) = 7.04, *p* < 0.04] and availability of technical and human resources [*F*(1,6) = 8.31, *p* < 0.03] were higher on day-shifts. A shift-phase × shift interaction for physical demands [*F*(1,6) = 10.75, *p* < 0.02] indicated that these demands were only on night-shifts perceived as being lower on the beginning (*M*= 1.67, SD = 0.10) than on the middle (*M*= 2.00, SD = 0.90; *p* < 0.002) and end of shift (*M*= 2.28, SD = 0.31; *p* < 0.07). No effect of shift-phase was observed for either job dimension, except for a marginal effect of co-worker support [*F*(1,6) = 5.74, *p* < 0.06], that was higher, though non-significantly, on shift-beginning (2.11) than on the rest of the shift (1.64 and 1.65 respectively).

**Table 2:** Operators’ job perception (*M* ± SD) on day-shifts and night-shifts, followed by the *p*-value of the ANOVA.

	Day-shifts	Night-shifts	*p*
Psychological demands	2.04 (0.18)	1.70 (0.17)	0.06
Skill expression	3.21 (0.20)	*2.75* (0.16)	0.001
Supervisor support	1.65 (0.17)	1.32 (0.16)	0.02
Co-worker support	2.14 (0.27)	1.46 (0.11)	0.04
Resource availability	3.34 (0.28)	3.00 (0.22)	0.01

– In addition, interesting relations occurred between the different dimensions of job perception, as indicated by significant positive correlations between perceived physical and psychological job demands on the beginning and end of day-shifts (respectively *r* = 0.74, *p* < 0.04; *r* = 0.75, *p* < 0.03), and on the middle and end of night-shifts (respectively *r* = 0.85, *p* < 0.007; *r* = 0.77, *p* < 0.03). On the beginning of day-shifts physical demands and resource availability were both associated with work–family interferences (respectively *r* = 0.78, *p* < 0.03; *r* = 0.79, *p* < 0.02). On this shift-phase, perceived autonomy showed a positive relation with skill expression (*r* = 0.72, *p* < 0.05) and a negative relation with work–family interferences (*r* = -0.73, *p* < 0.04). Further, on night-shifts, co-worker support was associated with physical and psychological job demands on shift-middle (respectively, *r* = 0.75, *p* < 0.03; *r* = 0.83, *p* < 0.01) and with supervisor support (*r* = 0.88, *p* < 0.003) on shift-end. On the latter shift-phase, both kinds of social support were also associated with resource availability (supervisor support: *r* = 0.82, *p* < 0.01; co-worker support: *r* = 0.93, *p* < 0.001). Furthermore a positive relation occurred between supervisor support and work–family conflicts on shift-middle (*r* = 0.86, *p* < 0.006) and shift-end (*r* = 0.82, *p* < 0.01).
– Correlation analyses between the different measures indicated that alertness was negatively correlated with response latencies in the working memory task (high external load) on the beginning of day-shifts (*r* = -0.75, *p* < 0.03), and positively with the perceived effort during task completion on the beginning of night-shifts (*r* = 0.81, *p* < 0.01), indicating that task performance increased with alertness. High alertness was also associated with low perceived physical job demands on the end of day-shifts (respectively, *r* = -0.77, *p* < 0.02) and with low psychological job demands on the beginning of night-shifts (*r* = -0.72, *p* < 0.04).

In summary, operators in the present experiment displayed higher alertness on shift-beginning than on later shift-phases, both on day-shifts and on night-shifts. This then indicates that a decreasing alertness profile across the shift is not limited to day-shifts. At the same time, operators perceived physical job demands as being significantly lower on the beginning of the day- and night-shifts compared to the remaining times of the shifts. This impression was confirmed by the finding of significant lower real job-activity on shift-beginning compared to shift-middle. In addition, negative correlations were observed between alertness and job-demands (end of day-shifts and beginning of the night-shifts). Taken together, these findings would then indicate that in this field study perceived and effective job demands were high when operators’ self-reported alertness was low. Hence, in the previous study high job demands were significantly associated with high cognitive performance and alertness on shift-beginning.

Thus, a different profile emerges for the two job-situations, indicating that finely tuned and temporally situated investigations of workload are most probably specific to a given job situation (Siegrist, 2010). This may be attributed to the characteristics of the work activity, with a high-load event systematically scheduled on shift beginning for air traffic controllers (control of a test flight), enabling anticipation of this workload as expressed by higher alertness and cognitive performance, as well as an association between task demands and resources in the work environment. In contrast, satellite control involved a supervisory activity all over the shift, and throughout the shift did operators perceive resources as being higher than job demands. According to Karasek’s model (Theorell and Karasek, 1996) satellite control thus could be characterized as a low-strain or relaxed job situation. Furthermore, both job activity (perceived job demands and real-job activity) and alertness were higher on day-shifts than on night-shifts. Correlatively, job resources were also perceived as being significantly higher on day-shifts. These results then stress the interest to take into account organizational factors, i.e., shift and shift-phase, in order to investigate in detail workload in a given job-situation.

Elsewhere, results obtained in the working memory task appeared to be contradictory, at least at first sight. Indeed, task performance increased across the shifts in agreement with decreased perceived task difficulty, however, the reported mental effort to perform the task increased across the shift. The former effects may be favored or generated by the procedure involving three repetitions of the task on a given shift. Though on each occasion items were presented in a different order it may not be excluded that decreasing response latencies and perceived task difficulty are the result of a learning process. Conversely, improved task performance could result from a more marked effort provided by operators while performing the task later on the shift, and this more especially as alertness decreased precisely by the end of the shift while physical work demands were increased. In this case, the results confirm previous studies reporting a significant relation between cognitive performance and alertness only in more demanding task conditions (Mélan et al., 2007; Galy et al., 2008, 2012).

## DISCUSSION

One of the major contributions of the field studies reported here is the finding of significant relationships between operators’ functional state (alertness) and workload (real-job activity, perception of work demands). Further, both perceived job-demands and job-resources were significantly higher on day-shifts than on night-shifts. These are important findings as they indicate that subjective measures like the results of the job environment questionnaire used in the present studies confirm the decrement of body functions typically reported during the night. It is now widely accepted that the biological constraints imposed during night-work may have deleterious effects on workers’ performance and health (Costa, 1996). Therefore the work organization may notably differ between the different shifts in a number of job-situations, including air traffic, hospital care …, by limiting the number of consecutive night-shifts, but also by reducing the staff members on duty during the night (decreased social support, control and resource availability), and the scheduled tasks on night-shifts in order to decrease employees’ workload (decreased perceived work demands; Cavallo et al., 2002).

A second important finding was the demonstration of an alertness decrease across day-shifts in air traffic controllers, and across day- and night-shifts in satellite controllers. Hence, alertness would be expected to increase across the day, as has been systematically reported in controlled laboratory conditions and several real-job settings (Folkard and Tucker, 2003; Akerstedt, 2007; Galy et al., 2008; Dawson, 2012). These findings then lend further support to previous studies suggesting that in some job situations at least operators may anticipate high demands on shift-beginning (Andorre and Quéinnec, 1998; Cariou et al., 2008). The findings that on shift-beginning alertness and working memory performance were highest in air traffic controllers correlatively to significant higher perceived psychological demands favor this interpretation. We suggest in agreement with model’s (Galy et al., 2012) that mental load measures most probably reflect some specific cognitive process involved in the task/work-activity a subject has to perform, whereas alertness would refer to mental resources available to perform a task (Wickens, 2008). Accordingly, if mental resources are low (at shift-end in the reported studies), then performance would also be decreased, what was indeed observed in the first experiment. Hence, while in the second experiment operators’ alertness was indeed positively associated with working memory performance, it was negatively correlated with job demands. From this point of view, the organization of control activities across the shift and the resulting and perceived job demands (highest in shift-middle) did not match the time-course of participants’ resources in this job situation. This kind of observations may then allow to organize job tasks in order to find a better match between job demands and resources issued from the participant (i.e., alertness for instance) and from the work environment. Alternatively, perceived co-worker support was higher on shift-beginning and may have influenced the perception of work demands in that operators perceived lower job demands on that shift-phase. The present data focus on perception of the work environment over a short time-period (i.e., 1 h prior rating the questionnaire), and might therefore be strongly dependent on the job-situation considered, as has been suggested by Siegrist (2010). Clearly, further investigations including a larger number of participants are necessary to elude these questions.

Elsewhere, dimensions or measures representing some effortful process (i.e., physical and psychological job-demands, work–family interferences and tension) were positively associated with each other and negatively with dimensions or measures that relate to resources in the work environment (i.e., technical and human resource availability, social support, and control). Conversely, those dimensions representing resources in the work environment were in turn correlated with each other. A coherent picture emerged from these findings and both job situations have been interpreted in line with the job-strain literature as low strain or passive/active job situations. The results raise the possibility that resources as defined in Karasek’s model (i.e., control and co-worker support) may possibly be extended to additional types of resources in the work environment (i.e., technical and human resource availability), as has been suggested by others (Bakker et al., 2005). Likewise, additional demands have been documented by the present studies, i.e., work–family conflicts, a central concept of Greenhaus and Beutell’ (1985) model.

Alertness has also been considered in the presents studies as an additional resource of an operator in her/his work environment. Theorell and Karasek (1996, p. 10) include alertness requirements on contrary among psychological job demands . “Psychological job demands, that measure mental workload and alertness requirements (but not physical demands), include qualitative but also quantitative demands of work loads and demands of interpersonal interactions.” Our data also stress the importance of physical demands in addition to psychological demands, as has been stressed previously by others (Roquelaure et al., 2007). More especially physical job demands may reflect fatigue, which has been shown elsewhere to be predicted by high work demands (Akerstedt et al., 2004). More generally, work stressors, including demand and autonomy, have been shown to be related to the frequency of occupational injuries and near-misses (Hemingway and Smith, 1999; Goldenhar et al., 2003).

## CONCLUSION

The tools developed for the field studies reported in the present contribution were derived from different research fields and included subjective and objective workload measures. Like other subjective tools, these questionnaires may more readily be used in field studies, are cheaper and less time-consuming than recordings with physiological devices. They enabled in particular a finely tuned description of workload on different shift-phases and on different shifts in two different job situations. They also provided a broader view of those features that may represent resources in a given work-situation. Moreover, the theoretical concepts developed in the job-strain models have been adequately applied to explore this more focal job-perception in specific work-situations.

It may, however, not be excluded that these tools did not provide an exhaustive picture of an individual’s resources at work. Indeed, it seems plausible to include in future studies additional dimensions, assessing in particular motivational aspects, but also job experience and age. Accordingly, investigations based on this methodology should enable defining more accurately demands in a person’s work environment, and allow prompting recommendations in order to organize tasks most efficiently according to the specificities of a given shift. This should allow meeting more accurately job demands by avoiding overload and underload across shift-phases, more especially in safety-related job-situations (Folkard and Tucker, 2003; Flatley et al., 2004; Folkard and Lombardi, 2006).

## Conflict of Interest Statement

The authors declare that the research was conducted in the absence of any commercial or financial relationships that could be construed as a potential conflict of interest.
